# Translation Elongation Factor eEF1A2 is a Novel Anticancer Target for the Marine Natural Product Plitidepsin

**DOI:** 10.1038/srep35100

**Published:** 2016-10-07

**Authors:** Alejandro Losada, María José Muñoz-Alonso, Carolina García, Pedro A. Sánchez-Murcia, Juan Fernando Martínez-Leal, Juan Manuel Domínguez, M. Pilar Lillo, Federico Gago, Carlos M. Galmarini

**Affiliations:** 1 Departamento de Biología Celular y Farmacogenómica, Pharma Mar S.A., Colmenar Viejo, Madrid, Spain; 2 Departamento de Química Física Biológica, Instituto de Química-Física “Rocasolano” (CSIC), Madrid, Spain; 3 Departamento de Ciencias Biomédicas, Unidad Asociada al IQM-CSIC, Universidad de Alcalá, Madrid, Spain

## Abstract

eEF1A2 is one of the isoforms of the alpha subunit of the eukaryotic Elongation Factor 1. It is overexpressed in human tumors and is endowed with oncogenic properties, favoring tumor cell proliferation while inhibiting apoptosis. We demonstrate that plitidepsin, an antitumor agent of marine origin that has successfully completed a phase-III clinical trial for multiple myeloma, exerts its antitumor activity by targeting eEF1A2. The drug interacts with eEF1A2 with a *K*_D_ of 80 nM and a target residence time of circa 9 min. This protein was also identified as capable of binding [^14^C]-plitidepsin in a cell lysate from K-562 tumor cells. A molecular modelling approach was used to identify a favorable binding site for plitidepsin at the interface between domains 1 and 2 of eEF1A2 in the GTP conformation. Three tumor cell lines selected for at least 100-fold more resistance to plitidepsin than their respective parental cells showed reduced levels of eEF1A2 protein. Ectopic expression of eEF1A2 in resistant cells restored the sensitivity to plitidepsin. FLIM-phasor FRET experiments demonstrated that plitidepsin localizes in tumor cells sufficiently close to eEF1A2 as to suggest the formation of drug-protein complexes in living cells. Altogether, our results strongly suggest that eEF1A2 is the primary target of plitidepsin.

The eukaryotic Elongation Factor 1A2 (eEF1A2) is one of the two isoforms of eEF1A in mammalian cells. Despite sharing 96% homology with the eEF1A1 isoform, expression of both proteins is mutually exclusive in different mammalian tissues, indicating that they should have distinctive functions. While eEF1A1 is present almost ubiquitously, eEF1A2 expression has been shown to be restricted to brain, muscles, heart, islet cells in the pancreas and endocrine cells in the gut of healthy individuals^1^. However, abnormal expression of this protein was recently found in many human tumors and transformed cell lines, including multiple myeloma and plasmacytoma cells as well as prostate, pancreatic, ovarian, breast, lung and liver cancers^1^,^2^. Although originally described as a translation factor necessary to deliver aminoacyl-tRNAs to the A site of the ribosome, it is now known that eEF1A2 also presents other non-canonical functions^3^. Indeed, eEF1A2 has been shown to have pro-oncogenic activities, such as inhibition of apoptosis^4^, control of unfolded protein degradation by the proteasome^5^, heat shock response^6^, actin bundling and cytoskeleton reorganization^7^ and regulation of oxidative stress^8^. Noteworthy, it was recently described that down-regulation of eEF1A2 with miR-663 in two pancreatic cancer cell lines reduced the level of the protein and attenuated their proliferation and invasion both *in vitro* and *in vivo*^9^.

Plitidepsin (Aplidin) is a cyclic depsipeptide originally isolated from the marine tunicate *Aplidium albicans*. It showed potent anticancer activity in preclinical assays as well as in several phase I/II clinical studies in humans^10^. Plitidpesin has recently completed a pivotal phase III clinical trial for multiple myeloma (clinicaltrials.gov identifier: NCT01102426) that has shown a statistically significant 35% reduction in the risk of progression or death over the comparator (p = 0.0054). This study has met its primary endpoint. In tumor cells, the compound induces a potent oxidative stress and, subsequently, a rapid and sustained activation of two members of the MAPK family: the serine/threonine kinases JNK and p38 (for a review see ref. 10). These events finally activate a caspase-dependent apoptotic cell death pathway. Nonetheless, and despite the current in-depth knowledge of these pharmacological activities, the primary biochemical target for plitidepsin is currently unknown. Of interest, in two different COMPARE analyses, plitidepsin showed no similarity with any other antitumor agent, possibly indicating that the drug is interacting with a novel target in cancer cells. The present work demonstrates that eEF1A2 is the primary target of plitidepsin.

## Results

### Sensitivity to plitidepsin is correlated with mRNA and protein expression levels of eEF1A2 in tumor cells

**Figure 1:**
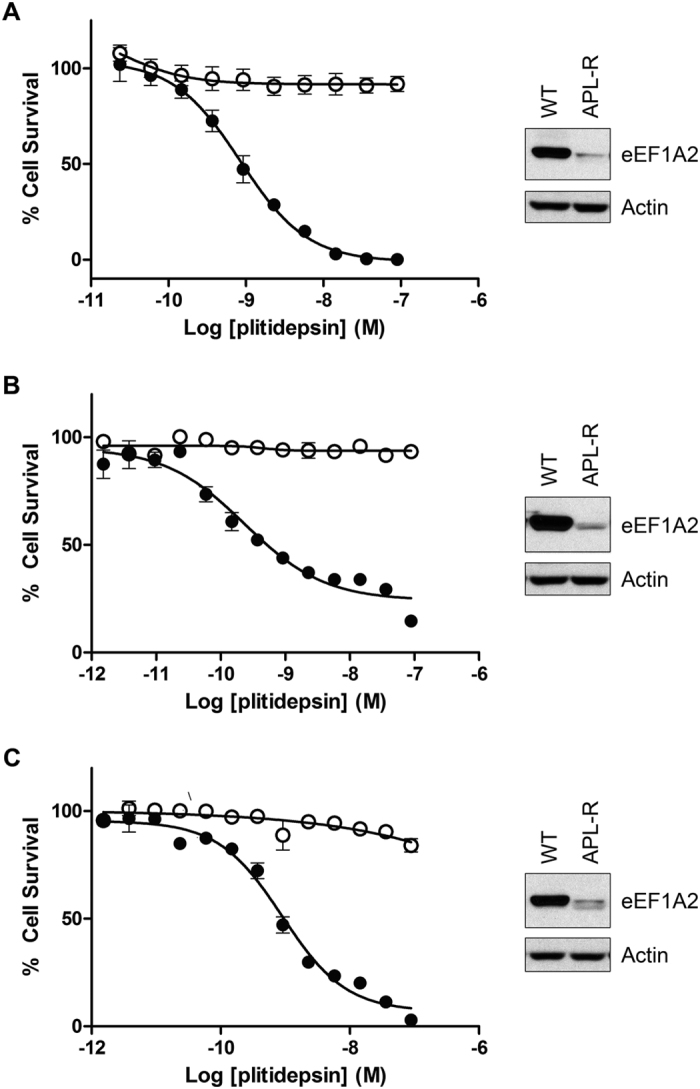
Plitidepsin-resistant cells lose expression of eEF1A2 protein. Cell growth inhibition curves were obtained after 72 h of exposure to several plitidepsin concentrations for HeLa and HeLa-APL-R cervical cancer cells (**A**), NCI-H460 and NCI-H460-APL-R non-small cell lung cancer cells (**B**) and HGC27 and HGC27-APL-R gastric carcinoma cells (**C**). In all cases eEF1A2 protein levels were analyzed by Western Blot as shown in the right hand side of each graph. ● wild type cells, ○ APL-R cells.

We have previously described that HeLa-APL-R cells are highly resistant to plitidepsin compared to their parental HeLa cells (IC_50_ values of >100 nM vs.1 nM) and that they do not show cross-resistance to other anticancer agents (Fig. 1A)^11^. To explore the molecular changes that could drive such a difference in sensitivity to plitidepsin between both cell lines, we firstly used Affymetrix HG-U133A microarrays. Table 1 shows the genes whose levels in HeLa-APL-R were down-regulated more than 2.5 times compared to the parental cells. Among them, eEF1A2 mRNA levels were approximately 2.75-fold lower in the resistant cells than in the wild-type line. We then investigated differences in protein expression between HeLa and HeLa-APL-R cells by Isobaric Tag for Relative and Absolute Quantitation (iTRAQ). Interestingly, the single protein with the highest difference of expression between both cell lines was eEF1A2 (Table 2). The reduced expression of eEF1A2 protein in HeLa-APL-R cells was confirmed by Western blot (right side of Fig. 1A).

Given the fact that eEF1A2 downregulation was the only major modification detected by both microarray and iTRAQ experiments, and to examine whether this was a general mechanism of plitidepsin resistance, we generated two additional plitidepsin-resistant cell lines from NCI-H460 (non-small cell lung cancer) and HGC27 (gastric carcinoma), as previously described for HeLa cells^11^. As shown in Fig. 1 (panels B and C), both NCI-H460-APL-R and HGC27-APL-R were highly resistant to plitidepsin as the drug did not cause any effect on cell growth at the highest concentration tested (100 nM) whilst the IC_50_ in the parental cells were 0.2 nM for NCI-H460 and 0.9 nM for HGC27. The resistance was specific for plitidepsin and not shared by any other anticancer agent and was not due to overexpression of efflux pumps (data not shown). We further analyzed eEF1A2 levels by Western blot and found that the resistant cell lines expressed very low levels of the protein, hardly detectable with this technique (right sides of Fig. 1B,C).

### Ectopic expression of eEF1A2 in HeLa-APL-R cells restores sensitivity to plitidepsin

**Figure 2:**
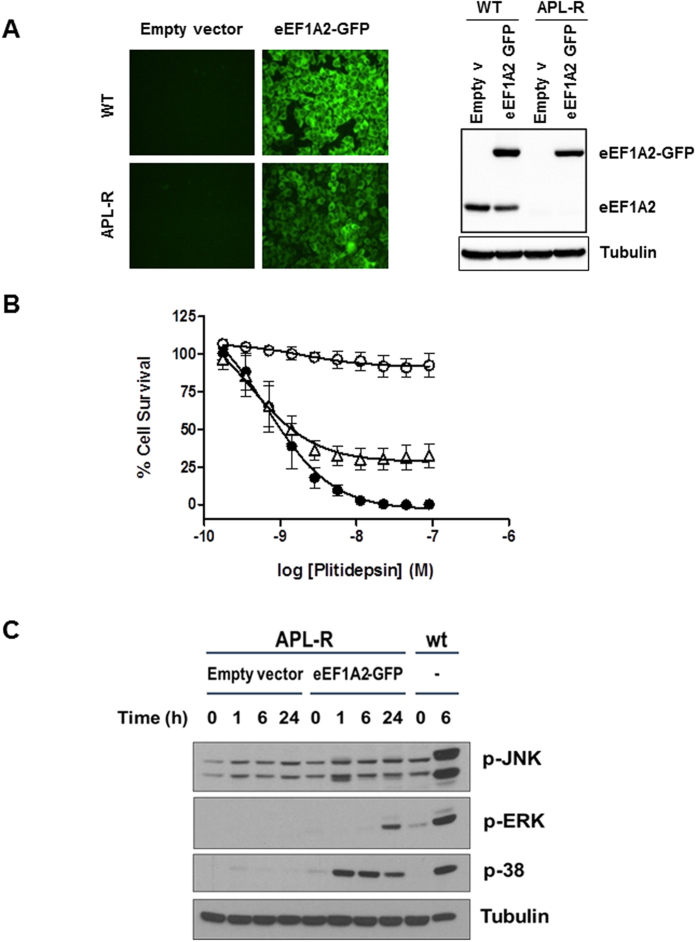
Ectopic expression of eEF1A2 in HeLa-APL-R cells restores sensitivity to plitidepsin. **(A)** HeLa and HeLa-APL-R cells were stably transfected with an expression vector encoding for eEF1A2-GFP fusion protein. Clones homogeneously expressing the GFP were selected. The levels of expression of eEF1A2 were analyzed by Western blot using specific antibodies. The position of the endogenous and the GFP-fusion proteins are indicated. HeLa-APL-R cells showed lower levels of eEF1A2 protein than HeLa cells. Levels of expression attained after transfection were similar to the endogenous levels. **(B)** Antiproliferative activity of plitidepsin in HeLa (●), HeLa-APL-R (○), or in the HeLa-APL-R subline stably transfected with a plasmid encoding eEF1A2-GFP (∆). Concentration-response curves were performed at 72 h. Cell growth was determined by the MTT method and expressed as percentage of control cell survival. **(C)** HeLa and HeLa-APL-R cells overexpressing eEF1A2-GFP were exposed to plitidepsin (450 nM) for the indicated times and protein expression analyzed by Western blot with the appropriate antibodies.

To explore whether the reduced levels of eEF1A2 in HeLa-APL-R cells were directly related to plitidepsin resistance, we stably transfected these cells with a plasmid encoding this isoform of the human elongation factor fused with GFP. Clones which ectopically expressed levels of the protein similar to those observed in parental HeLa cells (Fig. 2A) were then selected and subjected to cell growth inhibition experiments with plitidepsin. As shown in Fig. 2B, HeLa-APL-R cells stably transfected with eEF1A2-GFP partially recovered sensitivity to plitidepsin (Fig. 2B). In contrast, HeLa cells ectopically overexpressing eEF1A2-GFP did not show any additional sensitization to the drug (Supplementary Figure 1). Moreover, since HeLa-APL-R cells had completely lost the signaling events which are typically triggered by plitidepsin in the parental HeLa cells^11^, we checked whether the expression of eEF1A2-GFP in HeLa-APL-R had any effect on those events. As observed in Fig. 1C, such ectopic expression led to phosphorylation of p38, ERK and JNK when HeLa-APL-R cells were treated with plitidepsin. In agreement with the results in the antiproliferative experiments, HeLa cells overexpressing eEF1A2-GFP did not show any alteration in the signaling elicited by exposure to plitidepsin (Fig. 1C). Therefore we might conclude that eEF1A2 is directly involved in the mechanism of action of plitidepsin since its expression in resistant cells restores sensitivity to the drug.

### Plitidepsin interacts directly with eEF1A2 purified from rabbit muscle

**Figure 3:**
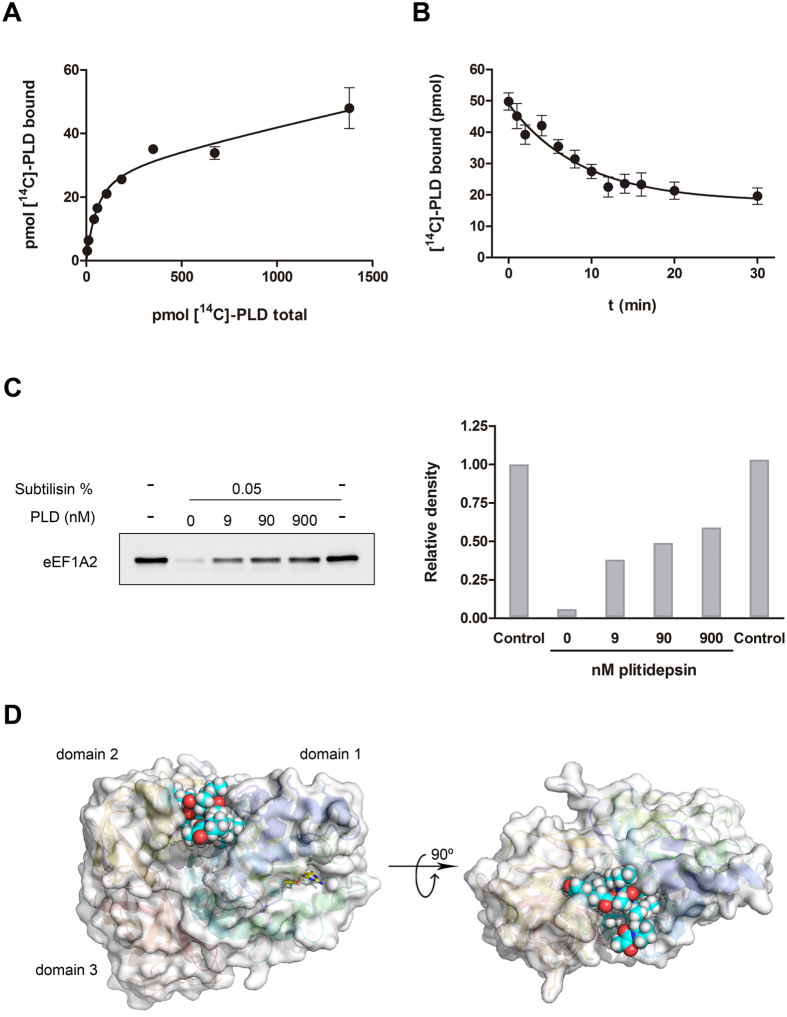
Interaction of [^14^C]-plitidepsin with eEF1A2 purified from rabbit skeletal muscle. **(A)** Saturation binding curve. Protein and radioligand were incubated for 1 h at room temperature and samples were processed as described in the text. Dots represent the mean of triplicate experiments with error bars representing S.D. The line shows the best fit to the mathematical equation derived by Swillens^12^ accounting for ligand depletion and non-specific binding. **(B)** Dissociation kinetics of [14C]-plitidepsin from eEF1A2. The experiment was performed as described in the text, and the experimental points were fitted to a single exponential decay equation by non-linear regression. Dots represent the mean of triplicate determinations with error bars denoting S.D., the line shows to the best fit to the exponential equation. **(C)** Plitidepsin protects eEF1A2 against proteolysis in DARTS assays. HeLa protein extracts were incubated with plitidepsin (APL) at the indicated concentrations for 1h. Extracts were then digested with the indicated concentrations of subtilisin for 30 min at room temperature. Samples were resolved by SDS-PAGE and the degradation of eEF1A2 analyzed by Western blot; quantitation of the bands from the DARTS assay was performed with the ImageJ software. **(D)** Molecular model showing the proposed mode of binding of plitidepsin (CPK) at the domain1-domain 2 interface of GTP-bound eEF1A2 (protein residues enveloped by a semitransparent solvent-accessible surface and GTP in domain 1 shown as sticks).

After having obtained evidence suggesting the involvement of eEF1A2 in the mechanism of action of plitidepsin, it was reasonable to consider this protein as a likely candidate to be the primary target of the drug. We then decided to check whether or not a direct interaction exists between plitidepsin and eEF1A2 and to quantify the affinity of such binding. To that end, eEF1A2 purified from rabbit muscle was used to run a saturation binding experiment with radiolabeled plitidepsin in the presence of a non-hydrolysable GTP analogue. Given the low specific activity of the radioactive compound, large concentrations of protein, similar to those of the ligand, had to be used to obtain a reliable signal by liquid scintillation counting and therefore ligand depletion had to be taken into account during data processing. Consequently, the mathematical approach described by Swillens^12^ was followed and, since the contribution of non-specific binding was already considered in the assumptions that gave rise to the descriptive equation of this approach, it was not necessary to include unlabeled plitidepsin in the experiment. This strategy allowed covering a wide range of radioligand concentrations, something unattainable due to solubility limitations if excess cold ligand had been included. As observed in Fig. 3A, the experimental data nicely fitted to the equation proposed by Swillens, rendering a *K*_D_ value of 79 ± 30 nM and a *B*_max_ of 0.7 mol per mol of protein. These results suggested that plitidepsin binds to a single site of rabbit muscle eEF1A2 with large affinity. The significant contribution of non-specific binding was clearly apparent at high radioligand concentrations, yielding a value of 0.014 ± 0.0005 for the “alpha” parameter corresponding to the ratio between non-specifically bound ligand and free ligand (i.e., the slope of non-specific binding in the graph).

To gain more details on this interaction, the kinetics of plitidepsin dissociation from eEF1A2 was measured by monitoring the time course of the displacement of prebound [^14^C]-plitidepsin by addition of a large excess of the unlabeled molecule. In these conditions, binding of the displaced radioligand was fully prevented; hence, the rate of disappearance of the protein-radioligand complex obeys single exponential decay kinetics^13^. As shown in Fig. 3B, the experimental data points nicely fitted the corresponding equation and therefore values of 1.9 ± 0.8 × 10^−3^ s^−1^ and 8.8 min could be determined for the first-order rate constant of dissociation, *k*_OFF_, and the target residence time, respectively.

To corroborate that plitidepsin also interacts with eEF1A2 in a cell extract, we took advantage of the DARTS (Drug Affinity Responsive Target Stability) technique^14^. To that end, we obtained a protein extract from HeLa cells and incubated it with plitidepsin at the indicated concentrations for 1 h. Each sample was then digested with subtilisin for 30 min at room temperature. Samples were resolved by SDS-PAGE and the degradation of eEF1A2 was analyzed by Western blot with the appropriate antibody. Plitidepsin protected eEF1A2 from digestion by subtilisin in a concentration-dependent manner, indicating that the compound was bound to the elongation factor (Fig. 3C). Taken together, all of these findings confirm that plitidepsin binds to eEF1A2, both as an isolated protein and in a cell lysate context, with high affinity.

In view of these results, we assessed the feasibility of complex formation between plitidepsin and eEF1A2 using molecular modelling. As a result of these studies, plitidepsin is proposed to bind at the interface between domains 1 and 2 of eEF1A2 where it would be stabilized by a large number of both polar and hydrophobic contacts (Fig. 3D). The former include several highly directional hydrogen bonds, namely, between (a) the hydroxyl group of Ist^1^ and the carboxylate of Asp35, (b) the carbonyl oxygen of Ist^1^ and ND1 of His295, (c) the carbonyl oxygen of Hip^2^ and the backbone amide nitrogen of Leu77, (d) the carbonyl oxygen of Thr^6^ and the guanidinium group of Arg37, and (e) the methoxy oxygen of Me_2_-Tyr^5^ and the hydroxyl of Ser291. Amongst the hydrophobic interactions, the isopropyl, pyrrolidine, and methoxyphenyl moieties of Leu^3^, Pro^4^, and Me_2_-Tyr^5^, respectively, are found to be in close contact with an apolar surface in domain 2 made up by the side chains of Tyr254, Ile256, Ile259, Val262, and Val264 whereas the isopropyl group of Hip^2^ is lodged in a cavity lined by the side chains of Trp78, Leu63, and Thr38 in domain 1. In this orientation the pyruvoyl group of plitidepsin is exposed to the solvent in a region close to the N-terminus of the highly charged second α-helix comprising from Lys36 to Lys51 and also to Lys255 from domain 2. Thus, the proposed plitidepsin-eEF1A2 complex (Fig. 3D) shows the drug snugly fitted at the interface between domains 1 and 2 of eEF1A2 in a location that, in eEF1A1, is known to lodge either the 3′ terminal adenine of all 20 aminoacylated tRNAs or the crucial Tyr162 of the guanine nucleotide exchange factors (GEF) eEF1Bα or eEF1Bδ, which catalyze the GDP for GTP exchange at the ribosomal A site.

### Plitidepsin interacts directly with eEF1A2 in a K-562 myelogenous leukemia cell lysate

**Figure 4:**
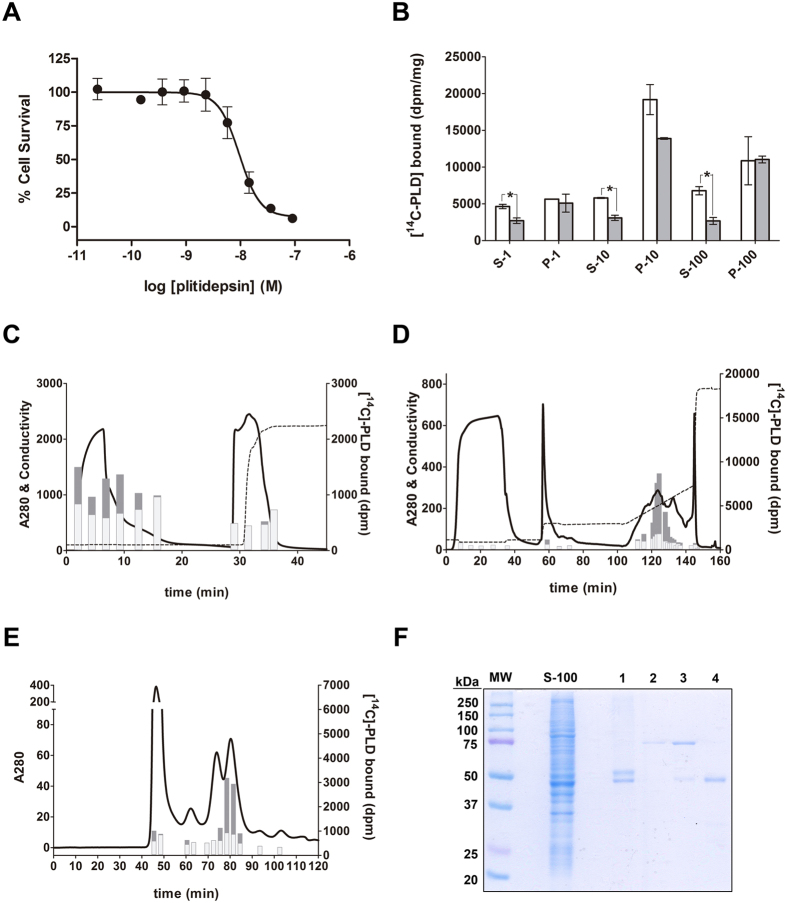
Identification of the plitidepsin-binding protein in a K-562 cells lysate. **(A)** Growth inhibition curve for K-562 chronic myelogenous leukemia cells after 72 h of exposure to several plitidepsin concentrations. (**B**) Subcellular fractions from K-562 cells were obtained and binding to [^14^C]-plitidepsin measured as detailed under “Materials and methods” using a 20-fold excess of unlabeled plitidepsin to determine the levels of non-specific binding (grey bars) that were compared to the levels of total binding (white bars) observed in the absence of unlabeled drug. In the horizontal axis “P” and “S” mean “pellet” and “supernatant”, respectively, with the figure aside denoting the relative centrifugal force (in thousands of g) used to obtain such fraction. Data (in dpm per mg of protein) represent the mean of three independent samples with error bars showing S.D. * means p < 0.05. **(C)** Anion-exchange chromatography on DEAE, flow rate 5 mL/min. **(D)** Cation-exchange chromatography on SP, flow rate 2.5 mL/min. **(E)** Size exclusion chromatography on Superdex 200, flow rate 4 mL/min. Representative fractions (4-mL) from each portion of the eluates were analyzed for their binding to [^14^C]-plitidepsin in the presence (grey bars, non-specific binding) or absence (black bars, total binding) of a 20-fold excess of unlabeled drug. Only fractions showing significant specific binding to the radioligand were pooled and progressed to the next chromatographic step. In all cases absorbance is expressed in mAU and conductivity in mS/cm. **(F)** PAGE-SDS of fractions from the size exclusion chromatography shown in panel D. MW, molecular weight markers; S-100 is the initial S-100 sample from the K-562 cells lysate; samples 1, 2, 3 and 4 correspond to the peaks eluting at 48, 63, 74, and 80 min respectively in the chromatogram displayed in panel E.

The dissociation constant described above is of the same order of magnitude as the plitidepsin concentrations that cause pro-apoptotic effects in tumor cells. This finding supports the hypothesis that eEF1A2 is a pharmacologically relevant target for the mechanism of action of this compound. However, other cellular targets could be capable of binding plitidepsin with similar, or higher, affinity. Consequently, a search was initiated to identify all the molecular entities with plitidepsin-binding ability in tumor cell lines. K-562 cells were selected to carry out this hunt given their ease to be cultured in large scale and their sensitivity to plitidepsin (IC_50_ = 9.35 nM, Fig. 4A). Cells were lysed and fractionated by differential centrifugation and the resulting individual fractions were tested for their ability to bind radiolabeled drug in a specific mode. Figure 4B shows that such an ability was retained by the soluble fractions whereas the associated pellets only bound [^14^C]-plitidepsin non-specifically. This was evidenced by the fact that a large excess of unlabeled drug did not cause a statistically significant reduction of the binding levels. The so-called “S-100” fraction (obtained after a 1 h centrifugation at 100,000 × g and mainly containing soluble cytosolic components) was then used to continue the fractionation by chromatographic methods.

With the aim of tracking all the components that showed plitidepsin specific binding and continuing their purification until their nature could be unequivocally ascertained, we used the same chromatographic strategy followed to purify eEF1A2 from rabbit muscle. A first step consisting of an anion-exchange chromatography showed that all the species capable of binding the drug eluted within the breakthrough (Fig. 4C). A subsequent cation-exchange chromatography step showed that the binding ability was widely spread throughout the eluate (Fig. 4D), although most of it was non-specific; only a few fractions eluting with a KCl-gradient showed significant specific binding to [^14^C]-plitidepsin. These fractions were then pooled and sieved through a size-exclusion chromatography column which yielded four major peaks (Fig. 4E): only the fourth one, eluting at 80 min (a position that corresponds to a MW of *ca*. 50 kDa), was able to bind plitidepsin specifically. When the peak was analyzed by PAGE-SDS (Fig. 4F), a band at *ca*. 50 kDa was observed, suggesting that a protein with that molecular weight was the major component of the peak and likely responsible for the ability to bind [^14^C]-plitidepsin. MALDI-TOF/TOF of the 50 kDa band after trypsin digestion demonstrated the presence of a peptide corresponding to fragment 85–96 of eEF1A2. Additionally, other peptides with masses corresponding to differential sequences in eEF1A1 (namely those corresponding to fragment 85–96 of Mr 1404.72, fragment 267–290 of Mr 2515.39 and fragment 396–423 of Mr 2995.40) were also detected, demonstrating that both isoforms were present in the peak. The rest of the peptides detected in this material corresponded to fragments whose sequences are common to both isoforms, thus discarding the presence of any other protein. It is therefore concluded that both isoforms of eEF1A are the only proteins present in K-562 cells that combine enough abundance and large affinity to be detected as capable of binding plitidepsin.

### Plitidepsin forms a complex with eEF1A2 first in the vicinity of the plasma membrane and then in the cytosol

We investigated whether a plitidepsin-eEF1A2 complex is formed in living cells by FLIM-phasor FRET methods. To that end, we first characterized the cellular distribution and fluorescence lifetime of eEF1A2-GFP (“Ac”) in HeLa and HeLa-APL-R cells stably transfected with such a fluorescent fusion protein. Supplementary Figure 2 shows the FLIM-phasor analysis of representative HeLa and HeLa-APL-R cells transfected with eEF1A2-GFP. The distribution of points in the phasor plot confirms a gradient distribution of eEF1A2-GFP inside the cells, with a constant lifetime *τ*_*GFP*_ of around 2.3 ns but variable autofluorescence. Specific eEF1A2-GFP fluorescence and autofluorescence intensities were calculated for each cell region from the positions on the line in the phasor plot connecting the Ac (green) and autofluorescence AF (grey) lifetimes (Supplementary Figure 2). When plitidepsin-DMAC was added to cell cultures, it was firstly detected at a very low concentration in the plasma membrane. After 30 minutes, a concentration gradient was reached inside each cell, with lower values in the vicinity of the plasma membrane and higher accumulations in specific intracellular regions (data not shown).

**Figure 5:**
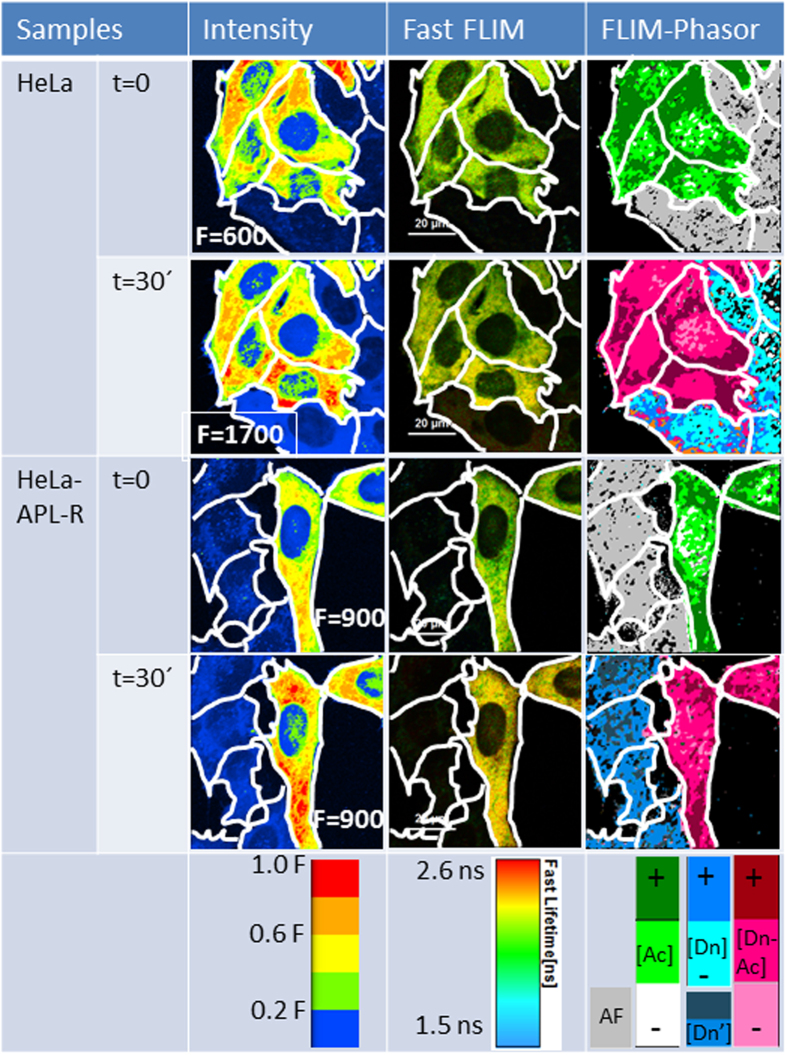
Cellular localization of plitidepsin-eEF1A2 complexes in living HeLa cells by steady-state fluorescence, fast FLIM and FLIM-phasor FRET imaging approaches. First column: Normalized steady-state fluorescence intensity images of representative HeLa and HeLa-APL-R cells transfected with eEF1A2-GFP, at time zero (t = 0) and after 30 minutes of treatment with 10 nM plitidepsin-DMAC, using a false five color intensity scale with F = maximum number of fluorescence photons per pixel. Acquisition time was 1.2 ms/pixel. Second column: Fast-FLIM images of the same cells from SymphoTime 64 Program (PicoQuant, GmbH), using a false rainbow scale (1.5–2.6 ns). Third column: FLIM-phasor images of the same cells from the SimFCS package. At time zero, only the Ac (eEF1A2-GFP) and autofluorescence (AF) signals are present. Grey represents AF; white, light green and dark green represent eEF1A2-GFP at low, medium and high concentration respectively; light and dark Cyan represent plitidepsin-DMAC species at low and high concentration, respectively in HeLa cells; Blue: plitidepsin-DMAC species in HeLa-APL-R cells; light, dark pink and garnet: plitidepsin-DMAC/eEF1A2-GFP FRET complexes.

Next, we used the FLIM-phasor FRET approach to detect complexes between plitidepsin-DMAC and eEF1A2-GFP in the plasma membrane and throughout the cytosol. Figure 5 shows fluorescence intensity, fast FLIM and FLIM-phasor images of representative groups of HeLa and HeLa-APL-R cells. Each image field from HeLa and HeLa-APL-R cells contains a mix of eEF1A2-GFP expressing and non-expressing cells. Cells expressing eEF1A2-GFP, either HeLa or HeLa-APL-R, show an important fluorescence intensity increase after 30 minutes of treatment with 10 nM plitidepsin-DMAC (Fig. 5, first column). Likewise, fast FLIM images show an increase in eEF1A2-GFP fluorescence lifetime in HeLa and HeLa-APL-R cells expressing the fusion protein (Fig. 5, second column). FLIM phasor analysis of the FRET-FLIM images (Fig. 5, third column) showed the formation of FRET complexes (marked with pink or garnet color in the Figure), both in HeLa and HeLa-APL-R cells expressing eEF1A2-GFP. The cyan/blue color in HeLa and HeLa-APL-R cells not expressing eEF1A2-GFP corresponds to “Dn” only phasors. FLIM-phasor FRET analyses presented here are compatible with high FRET efficiencies (*E* ~ 80–90%) from DMAC to GFP in plitidepsin-eEF1A2 complexes, both in HeLa and HeLa-APL-R cells over-expressing eEF1A2-GFP. Finally, the maximum concentration of plitidepsin-eEF1A complexes is observed in enriched eEF1A2-GFP regions.

## Discussion

Our data indicate that plitidepsin targets eEF1A2 in cancer cells. This protein is best known as one of the two isoforms of eEF1A that delivers aminoacyl-tRNAs (aa-tRNAs) to the A-site of the ribosome. eEF1A2 is a classic G-protein that can adopt two distinct conformations depending on whether GTP or GDP is bound in its catalytic domain, also known as domain I^15^. The GTP-bound conformation binds aa-tRNAs whereas it is the GDP-bound conformation that is involved in other functions such as enhancement of sphingosine kinase-1 (SK1) activity^16^. In the case of human phosphatidylinositol 4-kinase (PI4K) IIIB activation by eEF1A2, which results in increased cellular abundance of phosphatidylinositol 4-phosphate (PI4P), it is currently unknown which of the two forms is involved^17^. In contrast, both conformational states are known to interact similarly with actin, the filamentous form of which (F-actin) stimulates the rate of GTP hydrolysis, making use of the C-terminal 54 amino acids of domain III^7^. Indeed, the percentage of eEF1A that is associated with the actin cytoskeleton has been estimated at 70–90% depending on cell type^7^. These latter non-canonical pleiotropic functions clearly point to the oncogenic properties of eEF1A2^3^.

Until now, very few chemical entities have been identified as modulators of eEF1A2. Didemnin B and their close analogues tamandarins were among the first molecules described to interact with eEF1A. The original publication^18^ did not specify which isoform it was but since the protein utilized was isolated from rabbit reticulocytes one may think that it was eEF1A1. The reported *K*_D_ for such interaction was rather weak (200 μM for didemnin B binding to EF1A and 4 μM to the eEF1A-ribosome complex^18^). Other authors also reported the discovery of several flavonoids as well as phenanthridones (e.g. narciclasine, pancratistatine) that selectively bind to both eEF1A isoforms but none of these compounds has been tested in a relevant clinical settings yet^19^,^20^,^21^. More recently, the discovery of nannocystins as inhibitors of eEF1A1 has been disclosed^22^,^23^. These compounds showed promising potencies in cellular assays, with IC_50_ values reaching the sub-nM range, but their performance in *in vivo* models, including their safety profile, has not been disclosed yet. Our data obtained with several tumor cell lines support the hypothesis that eEF1A2 is the main target responsible for plitidepsin’s antiproliferative effects. Noteworthy, HeLa cells acquired resistance to the drug by decreasing the expression of eEF1A2 and sensitivity was significantly recovered by restoring eEF1A2 to normal levels. Likewise, signaling events making up plitidepsin’s signature in sensitive cells were restituted (at least partially) in resistant cells after eEF1A2 transfection. Similar findings were observed in two other plitidepsin-resistant cells, indicating that the resistance of HeLa-APL-R cells was not due to specific particularities of that cell line. In all cases, plitidepsin resistance was specific for the drug (and other members of the didemnin family) and not shared with any other anticancer drug family (data not shown).

Using biochemical approaches we confirmed that plitidepsin binds to eEF1A2 with a *K*_D_ of *ca*. 80 nM. The high affinity of this interaction is in consonance with the nanomolar antiproliferative potency of this compound, also observed in many previous reports (for a review see ref. 10). Likewise, from the measured value of the dissociation kinetic constant a residence time of circa 9 min is inferred for plitidepsin bound to the complex. This suggests that the dissociation of plitidepsin from eEF1A2 is moderately slow and, in addition to the pharmacodynamics benefits that a long residence time may provide, it is consistent with a high-affinity interaction. We ignore the reason for the discrepancy between these data and the modest affinity demonstrated by Ahuja and coworkers for didemnin B^18^. However, the values presented here are compatible with the potent antiproliferative effects of the drug, also observed in many previous reports^11^,^24^,^25^,^26^,^27^. Our microscopy data also support the direct interaction between plitidepsin and eEF1A2 in living cells. Using a FLIM-phasor FRET approach^28^,^29^, we detected complexes between plitidepsin-DMAC and eEF1A2-GFP, first in the plasma membrane and close to it, then distributed throughout the cytoplasm, and finally accumulated in well-defined perinuclear regions, following a pattern similar to that of eEF1A2 expression. Interestingly, our molecular model of the plitidepsin-eEF1A2 complex (Fig. 3D) shows the target protein in the GTP conformation, in agreement with the experimental data, and the drug docked into the same hydrophobic pocket of domain II that physiologically lodges either the terminal adenine of aa-tRNAs or the Phe/Tyr residue of eEF1Bα. This putative binding mode implies that plitidepsin can act as an effective competitor with both aa-tRNAs and eEF1Bα for binding to eEF1A2. In this regard, it is worth noting that the fast and complete apoptotic response elicited by plitidepsin in human leukemic cells at 10 nM was shown not to be affected by pretreatment with cycloheximide at concentrations that inhibit protein synthesis; instead, it could be prevented by pretreatment with the actin-binding agents cytochalasin B and jasplakinolide^30^, indicating that cell death induced by the drug is related to alterations of eEF1A function other than translation.

Since we identified the presence of eEF1A1 in the fraction binding [^14^C]-plitidepsin isolated from K-562 cell extract, the affinity of the molecule for such protein was also explored (Supplemental Figure 4) and we found that plitidepsin binds to eEF1A1 with a lower *K*_D_ than to eEF1A2 (180 and 80 nM for A1 and A2, respectively). Therefore, we can infer that the antiproliferative effects of the drug in tumor cells could not only be ascribed to its selectivity towards eEF1A2, but probably to the selective role of the latter with respect to eEF1A1 in promoting oncogenic transformations. The fact that plitidepsin-resistant cells only downregulate the expression of eEF1A2 favors this hypothesis. Notably, Soares and Abbott^31^ have reported significant differences among both isoforms in post-translational modifications clustering around the sequence variations sites. These differences may account for the divergence in biological roles observed for both proteins but should not be affecting the ability to bind plitidepsin. Indeed Panasyuk *et al*.^32^ have suggested that the higher tyrosine phosphorylation of eEF1A2 contributes to its ability to interact with the SH2 and SH3 domains of several signaling proteins playing critical roles in carcinogenesis. Such ability is not observed in eEF1A1, whose levels of tyrosine phosphorylation are lower and therefore this feature may provide eEF1A2 with greater potential to participate in oncogenic signaling pathways. On the other hand, the results of yeast two-hybrid experiments suggest that, contrary to eEF1A1, eEF1A2 may not be able to interact with the guanine nucleotide exchange factor EF1B^33^. Consequently nucleotide exchange in this latter isoform must be impaired as is its functionality in aminoacyl-tRNA delivery to the ribosome, a hypothesis that casts doubts on the actual role of eEF1A2 in translation when abnormally over-express in tumor cells. Indeed, when we studied the effect of EF1B on GDP/GTP exchange in eEF1A2 no significant increase of the nucleotide exchange rate was observed; likewise, the effect of plitidepsin on it was very modest (data not shown). Remarkably, it was previously published that didemnin B causes protein synthesis inhibition only at μM concentrations^34^ and we have observed similar results with plitidepsin^35^. Therefore, the possibility that translation inhibition may be responsible for the antiproliferative effect of the drug might be ruled out as already suggested by other authors^36^. In this respect, Potts and coworkers^37^ have recently corroborated the unlikelihood of such hypothesis for didemnin B.

Instead, we can imagine that one or more of the specific non-canonical functions of eEF1A2 could be affected by plitidepsin treatment. For example, it is known that eEF1A is able to directly increase the activity of sphingosine kinase-1 (SK1), an effect that is related to oncogenesis^16^,^38^. Preliminary results from our laboratory show that plitidepsin does, in fact, decrease the activity of this enzyme in tumor cells and this effect is not due to a direct effect of the drug on SK1 (data not shown). The hijacking of eEF1A with the consequent reduction of SK1 activity has already been described as a biological mechanism in dengue virus type-2 infected cells^39^. Another example is the specific binding of eEF1A2 to the i3 loop of M_4_, but not M_1_, muscarinic acetylcholine receptor^40^. This direct interaction with M_4_ regulates its cell membrane density and activity^41^. Using the PathHunter profiling technology (DiscoveRx, Fremont, CA, USA), we observed that plitidepsin does actually decrease acetylcholine-mediated CHRM4 activation by 55% in a CHO-transfected cell line while it did not affect CHRM1 (data not shown). Although preliminary, all of these results suggest that, by interacting with eEF1A2, plitidepsin could be affecting distinct functions of this protein in tumor cells. We can also speculate that the reported direct interaction of eEF1A2 with peroxiredoxin-1^8^ could also be perturbed by plitidepsin and this might be responsible for the potent oxidative stress that is observed after exposure of tumor cells to the drug. Finally, it was previously demonstrated that homodimeric eEF1A is required for the proteasomal degradation of ubiquitinated Nα-acetylated proteins. This is due to a specific interaction of eEF1A with aberrant polypeptides named defective ribosomal products (DRiPs) that are ligated to multi-ubiquitin chains following translation damage^42^; eEF1A also plays a role in sensing DRiP accumulation when the proteasome is inhibited^43^. Interestingly, the effects of plitidepsin in multiple myeloma cells were synergistic with bortezomib and with either thalidomide or lenalidomide^44^, two immune-modulatory drugs (IMiDs) that bind to the E3 ubiquitin ligase cereblon^45^.

The results presented in this work show that: i) there is a direct, high-affinity interaction of plitidepsin with eEF1A2; ii) such interaction has also been observed in living tumor cells using a FLIM-phasor FRET approach; iii) in K-562 cancer cell lysates, both eEF1A isoforms were the only proteins that combined enough abundance and affinity to be detected as capable of binding plitidepsin; and iv) sensitivity to plitidepsin in the different cancer cell lines studied here is dependent on eEF1A2 levels. Nonetheless, although these results point to eEF1A2 as the primary target of the drug, it must be acknowledged that still more work needs to be done to validate these findings in different models. For instance, it can be argued that more cell lines might have been used to extend these observations to a wider number of tumor cells of different origins and that alternative molecular biology techniques could have been applied to the resistant clones to validate the results presented here. In this regard, we did perform several silencing experiments to abrogate the expression of the EEF1A2 gene in HeLa cells but the results obtained were inconclusive because the large abundance of this protein prevented its complete silencing. Similarly, use of the CRISPR/CAS9 gene editing technology to knock-out the expression of eEF1A2 in human haploid cells did lead to increased resistance to plitidepsin compared to their parental counterparts (data not shown) but the high intrinsic rate of cell death precluded us from obtaining conclusive results. At any rate, new experiments are being conducted in new and different models to overcome these issues.

In our opinion, future research efforts must also concentrate on elucidating the mechanism(s) that link(s) binding to eEF1A2 to cell death. This knowledge will contribute to a better understanding of the effects caused by the drug and, in addition, will further expand our current understanding of the cellular functions of this moonlighting protein, potentially unveiling novel points for therapeutic intervention. From a more speculative perspective, this will also show whether the antiproliferative effect of this cyclic depsipeptide is only driven by its interaction with eEF1A2 or whether there are other indirect events that ultimately trigger apoptosis in cancer cells. For example, the relationship between oxidative stress, one of the main events involved in tumor cell death induced by plitidepsin, and its targeting of eEF1A2 is not fully understood yet. Whether this could be due to a direct effect on the protein or to the disruption of complexes of eEF1A2 with known (e.g. peroxiredoxin-1) or unknown proteins is currently under active investigation. In any case, the present results support the importance of eEF1A2 in the mechanism of action of a potent antitumor agent of marine origin that has successfully completed a phase III trial.

In summary, the results presented here demonstrate the critical role of the interaction between plitidepsin and eEF1A2 in its antiproliferative effect on tumor cells. More work is needed to ascertain the precise function(s) of eEF1A2 that is/are impaired by plitidepsin to cause tumor cell death so efficiently. At any rate, the present results demonstrate the importance of this protein in the mechanism of action of a potent antitumor agent of natural origin currently undergoing advanced clinical trials.

## Methods

### Reagents

Plitidepsin (C_57_H_87_N_7_O_15_, MW:1109.6, CAS No. 137219-37-5, APL), [^14^C]-plitidepsin (1.73 GBq/mmol), and a fluorescent coumarinated plitidepsin derivative (plitidepsin-DMAC) were prepared by PharmaMar (Colmenar Viejo, Spain). Stock solutions (1 mg/ml in DMSO) were prepared and stored at −20 °C. Complete (protease) and PhosStop (phosphatase) inhibitor cocktails were purchased from Roche Diagnostics (Mannheim, Germany). Anti-phospho-JNK (Thr183/Tyr185), anti-phospho-ERK1/2 (Thr202/Tyr204) and anti-phospho-p38 (Thr180/Tyr182) antibodies were purchased from Cell Signaling Technologies, Inc (Beverly, MA, USA). Anti-eEF1A and secondary HRP-conjugated goat anti-rabbit and goat anti-mouse antibodies were purchased from Santa Cruz Biotechnology (Santa Cruz, CA, USA). Anti-eEF1A2 (GTX102326) antibody was purchased from GeneTex (Irving, CA, USA). Anti-α-Tubulin (#T5168) antibody and subtilisin (EC 3.4.21.62) were purchased from Sigma-Aldrich, Inc. (St. Louis, MO, USA). A plasmid encoding eEF1A2-GFP (RG210716) was purchased from Origene (Rockville, MD, USA). Chromatography media (DEAE FF 16/10, SP HiPrep 16/10 and Superdex 200 16/600 columns) were obtained from GE Healthcare (Buckinghamshire, UK). All other reagents were from Sigma (St Louis, MO, USA).

### Cell techniques

HeLa cervix adenocarcinoma (ATCC CCL-2), K-562 chronic myelogenous leukemia (ATCC CCL-243), NCI-H460 lung cancer (ATCC HTB-177) and HGC27 gastric cancer (ATCC CRL-2506) cells were obtained from ATCC (Manassas, VA, USA) and cultured following standard procedures. Viability assays were performed utilizing 3-(4,5-dimethylthiazol-2-yl)-2,5-diphenyltetrazolium bromide (MTT) according to methods described elsewhere^46^. The stably plitidepsin-resistant HeLa cell subline (HeLa-APL-R) was generated at PharmaMar as previously described^11^ and the same protocol was used to generate other plitidepsin-resistant cell lines.

For transfection and selection of cell clones, HeLa and HeLa-APL-R cells were seeded in 24 well plates at 40% confluence and allowed to stand for 24 hours at 37 °C and 5% CO_2_. Before transfection, the culture medium was changed to Opti-MEM (Life Technologies, Carlsbad, CA, USA). Plasmids encoding eEF1A2-GFP were incubated with Lipofectamine (Life Technologies, Carlsbad, CA, USA) under the recommendations of the manufacturer and added to the cell medium. After 24 h incubation at 37 °C and 5% CO_2_, the culture medium was replaced by the standard DMEM supplemented with 2 mg/ml of G-418 for the selection of clones expressing the exogenous gene. Clones were further selected following the expression of the GFP-fused protein through fluorescence microscopy.

### Differential gene expression through DNA array

Total RNA was obtained from four samples of each HeLa and HeLa-APL-R cells with TRIzol reagent (Life technologies) under the recommendations of the manufacturer. Once purified, 15 μg RNA from each sample were reverse transcribed with a T7-Oligo(dT) promoter primer in the first-strand cDNA synthesis reaction, as indicated in the GeneChip One-Cycle Target Labeling Kit protocol (Affymetrix, Santa Clara, CA, USA). Second strand cDNA was then synthesized after eliminating the mRNA chain with RNase H. The double-stranded cDNA was then purified and served as the template for an *in vitro* transcription reaction in the presence of T7 RNA Polymerase and a biotinylated nucleotide analog/ribonucleotide mix for complementary RNA (cRNA) amplification and biotin labeling. The biotinylated cRNA targets were then cleaned up, fragmented, and hybridized to Human Genome U133A Arrays (Affymetrix, Santa Clara, CA, USA) during 16 h, using the GeneChip Hybridization, Wash and Stain Kit (Affymetrix, Santa Clara, CA, USA) following the manufacturer’s instructions. Then, arrays were washed and stained using the Fluidics Station 400 (Affymetrix, Santa Clara, CA, USA). Finally, arrays were scanned with a GeneChip Scanner 3000 (Affymetrix, Santa Clara, CA, USA). Data were subjected to quantile normalization to make them identical in statistical properties. Significance analysis of microarrays (SAM) was then applied to obtain the probe sets differentially expressed between HeLa and HeLa APL-R cells, establishing a Delta of 1.4 that gave a false discovery rate of 0.111. Tumor model gene expression profiles were analyzed by using Affymetrix U133 plus 2.0 arrays. The hybridizations were normalized by using the gc robust multichip averaging method from Bioconductor. eEF1A2 mRNA expression levels were determined by probe set “204540_at”.

### Isobaric Tag for Relative and Absolute Quantitation (iTRAQ) of differential protein expression

Protein extracts were obtained from HeLa and HeLa APL-R cells with lysis buffer (20 mM Tris-HCl (pH 7.5), 150 mM NaCl, 1% (v/v) Nonidet P-40, 2 mM EDTA, Complete and PhosStop cocktails) and kept on ice for 15 min. Cell extracts were cleared by centrifugation at 14,000 × g for 30 min at 4 °C. Proteins were then precipitated with trichloroacetic acid/acetone, washed with 6 volumes of acetone at −20 °C and dissolved in 100 μL of 0.5 M triethylammonium bicarbonate (TEAB) pH 8, 8 M urea. Protein was quantitated through the Bradford method and 110 μg of each sample were diluted up to 40 μL with of 0.5 M TEAB pH 8. Samples were reduced with 5 mM tris-(2-carboxyethyl)phosphine (TCEP) at 60 °C for 1 h and the cysteine-groups blocked with 10 mM methyl methanethiosulfonate (MMTS) at room temperature for 10 min. Samples were then diluted with 0.5 M TEAB pH 8 to bring the urea concentration below 2 M and digested with 10 μg of trypsin (Promega, Madison, WI, USA) at 37 °C overnight. Peptides were dried in the SpeedVac (Thermo Fisher Scientific, Waltham, MA, USA) and labeled with iTRAQ 8 plex, 3 h at room temperature after the protocol of AB SCIEX (Framingham, MA, USA). Samples were then pooled and 440 μg of protein subjected to IEF in a 13 cm Immobiline DryStrip pH 3-10NL (GE Healthcare, Piscataway, NJ, USA). The strip was then washed with water and cut into 25 pieces of 0.5 cm. Peptides from each of the pieces were then extracted, dried and suspended in 15 μL of 5% acetonitrile, 0.1% trifluoroacetic acid. A first chromatography was performed in an ETTAN LC (GE Healthcare, Piscataway, NJ, USA) with a reverse phase column Phenomenex Gemini 3 μm C18 110 Å. Samples were loaded in Buffer A, 20 mM triethanolamine in H_2_O, and eluted with a gradient of buffer B, 20 mM triethanolamine in acetonitrile, from 5 to 45%, at a flow rate of 150 nL/min. Fractions were collected and acidified. For the HPLC/MS/MS analysis, samples were loaded onto an Dionex PepMap C18 3 μm 100 Å column in buffer A, 0.1% formic acid in H_2_O, and eluted with a gradient from 5 to 90% of buffer B, 95% acetonitrile 0.1% formic acid, through a QSTAR ESI-QTOF (Life Technologies, Carlsbad, CA, USA). The combined information of the MS/MS was processed in MASCOT through the software Mascot Daemon (Matrix Science, Boston, MA, USA), using the database Sprot 20090603.

### DARTS assay

Protein extracts from HeLa cells were obtained by treating them with lysis buffer (see above) and kept on ice for 15 min. Cell extracts were cleared by centrifugation at 14,000 × g for 15 min at 4 °C. Protein extracts were then incubated with plitidepsin at the indicated concentrations for 1 h. Extracts were digested with the indicated concentrations of Subtilisin (EC 3.4.21.62) for 30 min at RT. Samples were resolved by SDS-PAGE and the degradation of eEF1A2 analyzed by Western blot. Quantitation of the eEF1A2 bands was performed with the Image Lab v5.2.1 software (Bio-Rad, Hercules, CA, USA).

### eEF1A2 purification and cellular fractionation

eEF1A2 was purified from rabbit muscle following the procedure described by Yaremchuk *et al*.^47^. The eEF1A2 concentration was determined spectrophotometrically using ε_280_ 45,380 M^−1^ cm^−1^ as deduced from its amino acid sequence (Swiss-Prot Q71V39). Regarding cellular fractionation, K-562 cells were grown in 10 L of DMEM medium supplemented with 10% (v/v) FBS, 100 units/mL penicillin, 0.1 mg/mL streptomycin, 1% (v/v) pluronic acid and 2 mM L-glutamine in a Wave bioreactor (GE Healthcare, Buckinghamshire, UK). When the culture reached a density of 1.5E6 cells/mL cells were harvested, washed twice with ice-cold PBS, and homogenized: typically 30 g of cell pellet was used for fractionation experiments. The resulting cell lysate was centrifuged at 1,000 × g for 10 min, the supernatant centrifuged again at 10,000 × g for 20 min and the newly obtained supernatant was finally centrifuged at 100,000 × g for 60 min. Pellets were resuspended in the same homogenization buffer using 1/20 of the volume of the original sample, and aliquots from the supernatants were withdrawn for analysis in binding assays. The soluble fraction resulting from the last centrifugation was equilibrated in 30 mM potassium phosphate pH 7,5; 1 mM magnesium chloride; 15% (v/v) glycerol and 6 mM β-mercaptoethanol, using a Sephadex G-25 column and it was then processed through several chromatographic steps similar to those followed during eEF1A2 purification, as detailed in the “Results” section. 4 mL fractions were collected and their ability to bind [^14^C]-plitidepsin was determined as described below.

### Binding assays

All samples in binding assays were tested in triplicate. For the saturation binding experiment, 100 nM rabbit eEF1A2 was mixed with several concentrations of [^14^C]-plitidepsin (ranging from 0.1 to 4 μM) in 45 mM Hepes-KOH pH 7.5, 5 mM magnesium acetate, 75 mM potassium chloride, 1 mM DL-dithiothreitol and 5% (v/v) DMSO in the presence of 1 μM Gpp(NH)p in a final volume of 500 μL. After 1 h incubation at room temperature, 450 μL were withdrawn, filtered through GF/C filters (Millipore, Bedford, CA) and washed three times with the same buffer used for the incubation. Filters were then removed, dried and their radioactivity was finally counted as a measurement of total (specific and non-specific) bound drug. An aliquot from each of the [^14^C]-plitidepsin solutions used to prepare each sample was counted in triplicate to determine the actual amount of total radioligand present in each case and such value was considered for data processing. Scintillation counting was transformed into concentration values considering the specific activity of the radioligand and the sample volume. The concentration of bound radioligand was related to the total amount of radioligand in the sample using the expression derived by Swillens^12^ to account for ligand depletion when the concentration of protein was similar to that of radioligand:


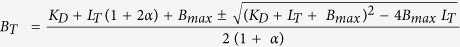


where B_T_ is the concentration of total (specific and non-specific) bound plitidepsin, L_T_ is the total concentration of plitidepsin in the sample, *K*_D_ is the dissociation constant, *B*_max_ is the maximum amount of drug bound to the protein and α is a parameter corresponding to the ratio between non-specifically bound ligand and free ligand (which in fact accounts for the dependency of non-specific binding with ligand concentration).

For dissociation kinetics, 1 μM [^14^C]-plitidepsin was mixed with 100 nM rabbit eEF1A2 in the same buffer as above for 1 h and either DMSO to 1% or unlabeled plitidepsin to reach 10 μM in 1% DMSO were added to the mixture whose final volume was 500 μL. At the selected times after this last addition, 400 μL of each sample were withdrawn and processed as above.

For the fractionation process, tested samples (fractions from either the subcellular fractionation or the chromatography eluates) were mixed with 500 nM [^14^C]-plitidepsin in 45 mM Hepes-KOH pH 7.5, 5 mM magnesium acetate, 75 mM potassium chloride, 1 mM DL-dithiothreitol and 5% (v/v) DMSO in the presence or absence of 10 μM plitidepsin, in a final volume of 500 μL. After 1 h incubation at room temperature 400 μL of each tube were withdrawn and processed as described above.

### FLIM-phasor FRET quantitation of eEF1A2-GFP/Plitidepsin complex formation *in vivo*

Two-photon fluorescence-lifetime imaging FRET microscopy following the phasor approach (FLIM-phasor FRET) was performed to demonstrate the interaction between eEF1A2 and plitidepsin in the cell. Experiments were performed on a MicroTime 200 system (PicoQuant, Germany) coupled with an Olympus IX71 inverted microscope. FLIM images were acquired with a single-photon avalanche diode (τ-SPAD, PicoQuant, Germany) and FF01-520/35 bandpass filter (Semrock, Germany). For FLIM-FRET measurements, HeLa and HeLa-APL-R cells stably transfected with eEF1A2-GFP (FRET Acceptor, “Ac”) were cultured in LabTek-II chambered coverglass slides (Thermo Scientific-Nunc), and treated at 37 °C with plitidepsin-DMAC (FRET Donor, “Dn”), keeping DMSO lower than 0.5% v/v.

FRET efficiencies were estimated using the FRET calculator tool included in program SimFCS (Laboratory for Fluorescence Dynamics, Irvine, CA), taking into account the contribution of “Dn” bleed-through (<10% in our experimental conditions) in the “Ac” channel, “Dn” unquenched by FRET, unbound “Ac”, direct “Ac” excitation and autofluorescence “AF” lifetime species present in each pixel. In our experimental conditions the “Ac” GFP can be excited directly by the laser pulse and by energy-transfer from plitidepsin-DMAC. FRET “Dn” and “Ac” trajectories on the phasor plot are curved and they represent realizations of all possible donor phasors quenched by FRET with different efficiencies (see Supplementary Figure 3). When plitidepsin-DMAC and eEF1A-GFP molecules are within FRET distances (less than 100 Å), the fluorescence intensity and lifetime of plitidepsin-DMAC should decrease from *τ*_*D*_ (“Dn” unquenched) to *τ*_*DA*_ (“Dn” quenched by the presence of the acceptor GFP) as a function of the FRET efficiency (E): *τ*_*DA*_ = *τ*_*D*_ · (1 − *E*). In contrast, both the intensity and the average lifetime of eEF1A2-GFP should increase.

### Molecular modelling

The previously reported three-dimensional structure of the complex formed between didemnin B and elongation factor eEF1A1 in the GTP conformation^48^ deposited in the Protein Data Bank under accession code 1SYW was employed as a template to generate a working model of human eEF1A2 using the one-to-one threading method implemented in the Phyre2 server. Plitidepsin contains a depsipeptide ring structure that resembles a twisted figure “8” and is made up by the linking of isostatine (Ist^1^), 2-(*R*-hydroxyisovaleryl) propionate (Hip^2^), leucine (Leu^3^), proline (Pro^4^), N,O-dimethyltyrosine (Me_2_Tyr^5^), and threonine (Thr^6^), all of them in the L configuration. The linear peptide attached to this latter amino acid contains N-methyl-D-leucine (MeLeu^7^) and pyruvoylproline (Pro^8^-Pyr^9^). Plitidepsin was docked into eEF1A2 by performing a best-fit superimposition on the previously reported eEF1A1-bound conformation of didemnin B^48^.

## Additional Information

**How to cite this article**: Losada, A. *et al*. Translation Elongation Factor eEF1A2 is a Novel Anticancer Target for the Marine Natural Product Plitidepsin. *Sci. Rep*. **6**, 35100; doi: 10.1038/srep35100 (2016).

## Supplementary Material

Supplementary Information

## Figures and Tables

**Table 1:** Comparative gene expression between HeLa wt and HeLa APL-R cells.

Gene	wt/APL-R ratio*
OPN3	2.52
IFIT1 (ISG54)	2.53
TNFRSF11B	2.56
TGM2	2.57
PALM	2.59
ADRBK2	2.60
CA9	2.68
eEF1A2*	2.75
PCSK1	2.76
FGG	2.77
PTGS1	3.28
SERPINE2	3.50
A2M	3.95
SP100	4.02
VPS13D	4.02
INHBB	4.09
SRGN	4.25
FN1	4.39
IFIH1 (MDA5)	4.39
SEPP1	6.14
TGFBI	6.49
COL4A6	8.37
IGLλ	9.53
SRGN	13.58
LUM	99.17

*mRNA from HeLa and HeLa APL-R cells were hybridized with HG-U133A Arrays (Affymetrix), scanned and data subjected to quantile normalization. SAM algorithm was applied to obtain the probe sets differentially expressed between HeLa and APL-R cells. Establishing a Delta of 1.4, false discovery rate is 0.111. wt/APL-R is the ratio between the mRNA levels of HeLa and APL-R cells.

**Table 2:** Comparative protein expression between HeLa and HeLa APL-R cells.

Protein	wt/APL-R* ratio
NALCN	3.48
TIM50	4.3
CD47	4.31
eEF1A2*	8.04

Protein extracts from HeLa and HeLa-APL-R cells were digested with trypsin and the resulting peptides labeled with iTRAQ 8 plex. Samples were then fractionated by isoelectrofocusing and reverse phase chromatography, and finally subjected to HPLC/MS to identify proteins differentially expressed between both cell lines. Proteins overexpressed more than 3 times are included.
