# Supplementary material for: Translation Elongation Factor eEF1A2 is a Novel Anticancer Target for the Marine Natural Product Plitidepsin
Source: Sci Rep. 2016 Oct 7;6:35100. doi: 10.1038/srep35100 (PMC5054363; doi:10.1038/srep35100)
Supplement: Supplementary Information [file srep35100-s1.pdf]

TRANSLATION ELONGATION FACTOR EEF1A2 IS A NOVEL ANTICANCER TARGET FOR THE MARINE NATURAL PRODUCT PLITIDEPSIN

Alejandro Losada[1], María José Muñoz-Alonso[1], Carolina García[2], Pedro A. Sánchez-Murcia[3], Juan Fernando Martínez-Leal[1], Juan Manuel Domínguez-Correa[1], Pilar Lillo[2], Federico Gago[3], Carlos M. Galmarini[1]

[1] Departamento de Biología Celular y Farmacogenómica, Pharma Mar S.A., Colmenar Viejo, Madrid, Spain

[2] Departamento de Química Física Biológica. Instituto de Química-Física “Rocasolano” (CSIC), Madrid, Spain.

[3] Departamento de Ciencias Biomédicas, Unidad Asociada al IQM-CSIC Universidad de Alcalá

SUPPLEMENTAL FILE

Supplemental material and methods

Mass spectrometry analysis of protein spots

Proteomic analyses by mass spectrometry were carried out in the Proteomics Facility of Universidad Complutense de Madrid, member of the ProteoRed network. The gel bands of interest were manually excised from gels for protein identification. Proteins selected for analysis were in-gel reduced, alkylated and digested with trypsin (12.5 ng/µL) according to common procedures. After digestion, the supernatant was

collected and 1 µL was dry-droplet spotted onto a MALDI target plate with 0.5 µL of 3 mg/mL of α-cyano-4-hydroxy-cinnamic acid matrix (Sigma) in 50% acetonitrile. MALDI-TOF MS analyses were performed in a 4800 Plus Proteomics Analyzer MALDI-TOF/TOF mass spectrometer (Applied Biosystems, MDS SCEX, Toronto, Canada) operating in positive reflector mode. All mass spectra were calibrated internally using peptides from the auto digestion of trypsin. MS/MS sequencing analyses were performed using collision-induced dissociation (CID). Peak list (m/z) from Data spectra were utilized to search Swissprot DB with no taxonomy restriction using MASCOT 2.3 through the Global Protein Server v 3.6 from ABSCIEX: positive matches were those yielding probability scores greater than the score fixed by MASCOT as significant with a p-value lower than 0.05. Finally, a targeted-proteomic approach was performed for detection of specific peptides in a 5500 Q-TRAP ABSCIEX mass spectrometer coupled to an Eksigent nano-HPLC system. Skyline (64-bit) version 1.6 software was used to build and optimize the multiple reaction monitoring (MRM) acquisition method and to ensure correct peak detection by manually inspection of the data.

LEGENDS TO SUPPLEMENTARY FIGURES

Supplementary Figure 1. Antiproliferative activity of plitidepsin in HeLa (●), HeLa-APL-R (○), or in a HeLa subline stably transfected with a plasmid encoding eEF1A2 (▲).

Supplementary Figure 2. FLIM-phasor characterization of eEF1A2-GFP in living HeLa and HeLa-APL-R cells. (A) The shape of the distribution of points in the phasor plot confirms a gradient distribution of eEF1A2-GFP (FRET "Ac"). The upper left side is dominated by eEF1A2-GFP (dark brown), and the right down side mainly consists of autofluorescence AF (grey). In any given pixel, mixture of GFP and AF must be on the green line. Fractional intensities of eEF1A2-GFP and AF in each pixel were calculated from the corresponding positions of the phasors on the green line in the phasor plot connecting the lifetimes of pure eEF1A2-GFP and autofluorescence. **(B)** Phasor color maps of the FLIM image. The colors of pixels correspond to the clusters of eEF1A2-GFP at different concentrations identified in the phasor plot.

Supplementary Figure 3. FRET efficiency estimation from FRET "Dn" and FRET "Ac" trajectories. (A) Phasor plot of the FLIM images of a representative group of HeLa WT and HeLa eEF1A2-GFP cells untreated, and at different times (0, 11, 21 and 30 min) of incubation with 10 nM plitidepsin-DMAC. Clusters of phasors corresponding to the different molecular species are identified in the phasor plot with different colors. Grey: autofluorescence AF; white, light green and dark green: eEF1A2-GFP (FRET "Ac") at low, medium and high concentration respectively; light and dark cyan: plitidepsin-

DMAC *Species B* (FRET "Dn") at low and high concentration, respectively; orange: plitidepsin-DMAC *Species C*; light, dark pink and garnet: plitidepsin-DMAC/eEF1A-GFP complexes **(B)** First row: Contour phasor plots of the FLIM images of the same cells at different times, including simulated FRET "Dn" (red curved line) and FRET "Ac" (blue) 0-100% FRET efficiency trajectories on the phasor plot, from the FRET calculator tool included in SimFCS program (Laboratory for Fluorescence Dynamics, Irvine, CA). Second row: Enlarged contour phasor plots of FRET "Ac" only (t=0) and FRET "Dn-Ac" (t=30') FLIM images including the color cursors used for the FRET efficiency estimation: blue: FRET "Dn", τ_{DMAC}=2.6 ns; red: FRET "Ac", τ_{GFP}=2.3 ns; pink: FRET "Dn-Ac", with FRET efficiency of about 80%. Third row: overlay of FLIM-phasor and fluorescence intensity images of the same group of cells, treated with 10 nM plitidepsin-DMAC (t=30'), showing the cellular concentration distribution of plitidepsin-DMAC/eEF1A2-GFP complexes (light, dark pink and garnet). Note that the edges of the pink FRET regions (white line) co-localize with the cell plasma membrane.

Supplementary Figure 4. Saturation binding of [^{14}C]-plitidepsin to eEF1A1. eEF1A1 was purified from rabbit liver as described in (48). Protein and radioligand were incubated for 1 h at room temperature and samples were processed as described in the text for EF1A2 and [^{14}C]-plitidepsin. Dots represent the mean of triplicate experiments with error bars representing S.D. The line shows the best fit to the mathematical equation derived by Swillens (21) accounting for ligand depletion and non-specific binding, yielding K_D 183±32 nM.

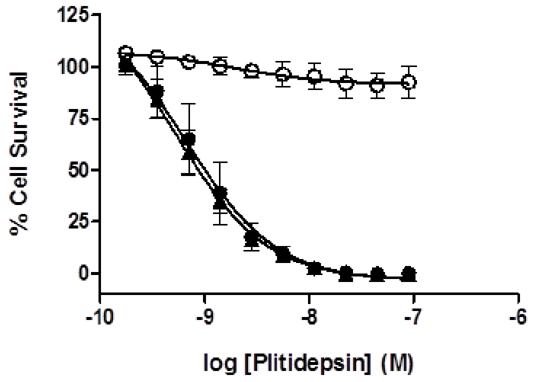

125
100
75
50
25
0
% Cell Survival
-10
-9
-8
-7
-6
log [Plitidepsin] (M)

HeLa eEF1A2-GFP ("Ac" only)

A)

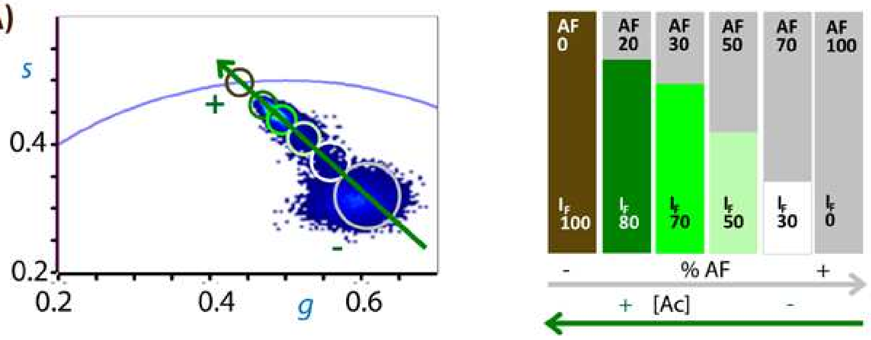

B)

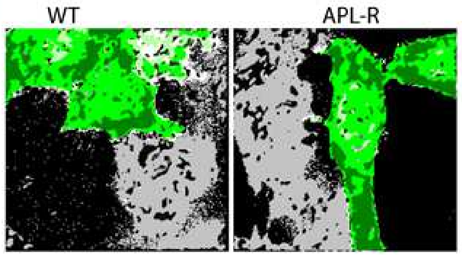

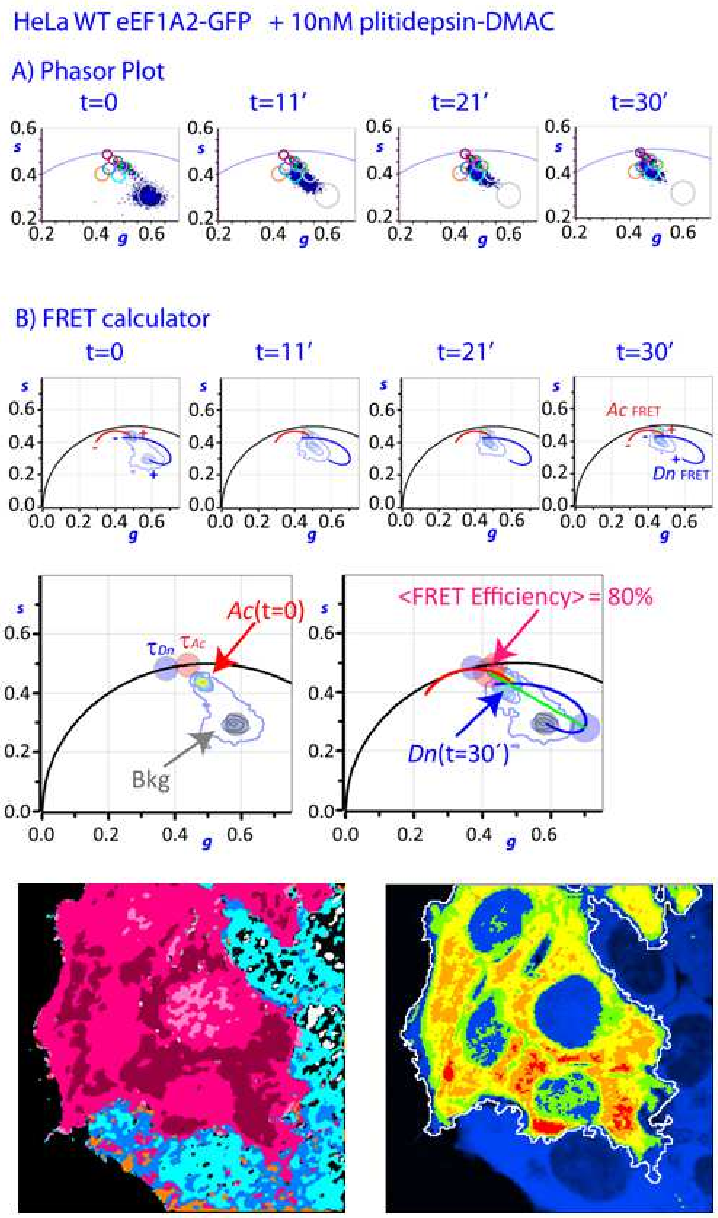
HeLa WT eEF1A2-GFP + 10nM plitidepsin-DMAC
A) Phasor Plot
t=0
t=11′
t=21′
t=30′
B) FRET calculator
t=0
t=11′
t=21′
t=30′
Ac FRET
Dn FRET
Ac(t=0)
τDn
τAc
Bkg
<FRET Efficiency>= 80%
Dn(t=30′)

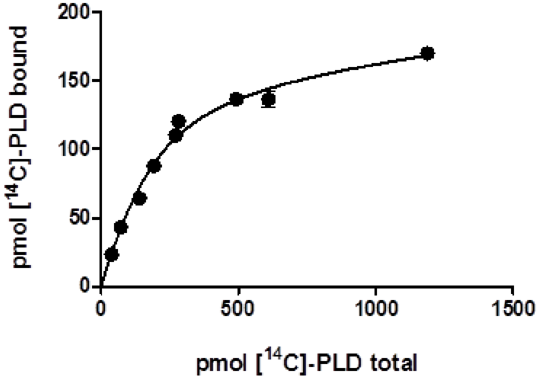

200
150
100
50
0
0
500
1000
1500
pmol [14C]-PLD bound
pmol [14C]-PLD total
